# Linear accelerator maintenance cost analysis

**DOI:** 10.1002/acm2.14246

**Published:** 2023-12-22

**Authors:** Marco Carlone, Wayne Beckham, Cheryl Duzenli, Kirpal Kohli, Scott Tyldesley

**Affiliations:** ^1^ Department of Medical Physics BC Cancer – Kelowna Kelowna BC Canada; ^2^ Department of Medical Physics BC Cancer – Victoria Victoria BC Canada; ^3^ Department of Physics University of Victoria Victoria BC Canada; ^4^ Department of Medical Physics BC Cancer – Vancouver Vancouver BC Canada; ^5^ Department of Surgery University of British Columbia Vancouver BC Canada; ^6^ Department of Medical Physics BC Cancer – Surrey Surrey BC Canada; ^7^ Department of Radiation Oncology BC Cancer – Vancouver Vancouver BC Canada

**Keywords:** linear accelerator, linear accelerator components, linear accelerator maintenance cost analysis, maintenance costs, service cost ratio

## Abstract

**Purpose:**

Medical linear accelerators are the most costly standard equipment used in radiation oncology, however the service costs for these machines are not well understood. With an increasing demand for linear accelerators due to a global increase in cancer incidence, it is important to understand the expected maintenance costs of a larger global installed base so that these costs can be incorporated into budgeting. The purpose of this investigation is to analyze the costs for medical linear accelerator service and maintenance at our institution, in order to estimate the service cost ratio.

**Methods:**

We collected the costs of parts used for all service work done on 32 medical linear accelerators over a two year period. The data was segregated by center, machine, linear accelerator type, and failure area in the machine.

**Results:**

We found the service cost ratio (excluding software support expenses) to be 3.13% [2.74%, 3.52%,]. We observed a variability of parts costs, and overall variability of the service cost ratio to be between 2.14% and 5.25%. This result is lower than other estimates for service costs for medical equipment in general and medical linear accelerators specifically. Two‐thirds of the service costs were due to labor costs, which indicate the importance of a well‐trained service technician workforce.

**Conclusions:**

We estimated the service cost ratio for medical linear accelerators to be 3.13% [3.52%, 2.74%] of the initial capital cost. This result was lower than other estimates of the service cost ratio.

## 1 INTRODUCTION

Medical linear accelerators are the most expensive commonly used equipment in Radiation Oncology. In 2023, new linear accelerator equipment capable of Volumetric Modulated Arc Therapy (VMAT) and Image Guided Radiotherapy (IGRT) can have costs upwards of $4 M USD. Including the capital cost of treatment rooms with sufficient shielding needed for radiation protection, costs for a new facility can easily reach many million US dollars. Beyond the startup purchasing and construction costs, there are significant costs associated with maintaining a radiotherapy facility in terms of repairing and keeping medical linear accelerators operationally safe.

The maintenance costs of medical linear accelerators, and medical equipment generally, are difficult to estimate. The typical metric for reporting medical equipment maintenance costs is to express annual cost including labor, parts, contracts, insurance, rents, etc. as a percentage of the initial equipment cost. In the United States, this ratio can very between 5−10%.^1^ It is difficult to obtain recent data about this ratio. In an older study of 19 large hospitals this ratio was found to be 7.4%, however, the authors indicated that there was large variability.^2^ In a case study for a single large institution, maintenance cost ratio was 4.36%.^3^ The value of these results is difficult to assess since these studies were done some time ago in a different era of medical services delivery, however, we include them here to provide some baseline expectations of what service cost ratio can be in a medical setting.

**FIGURE 1:**
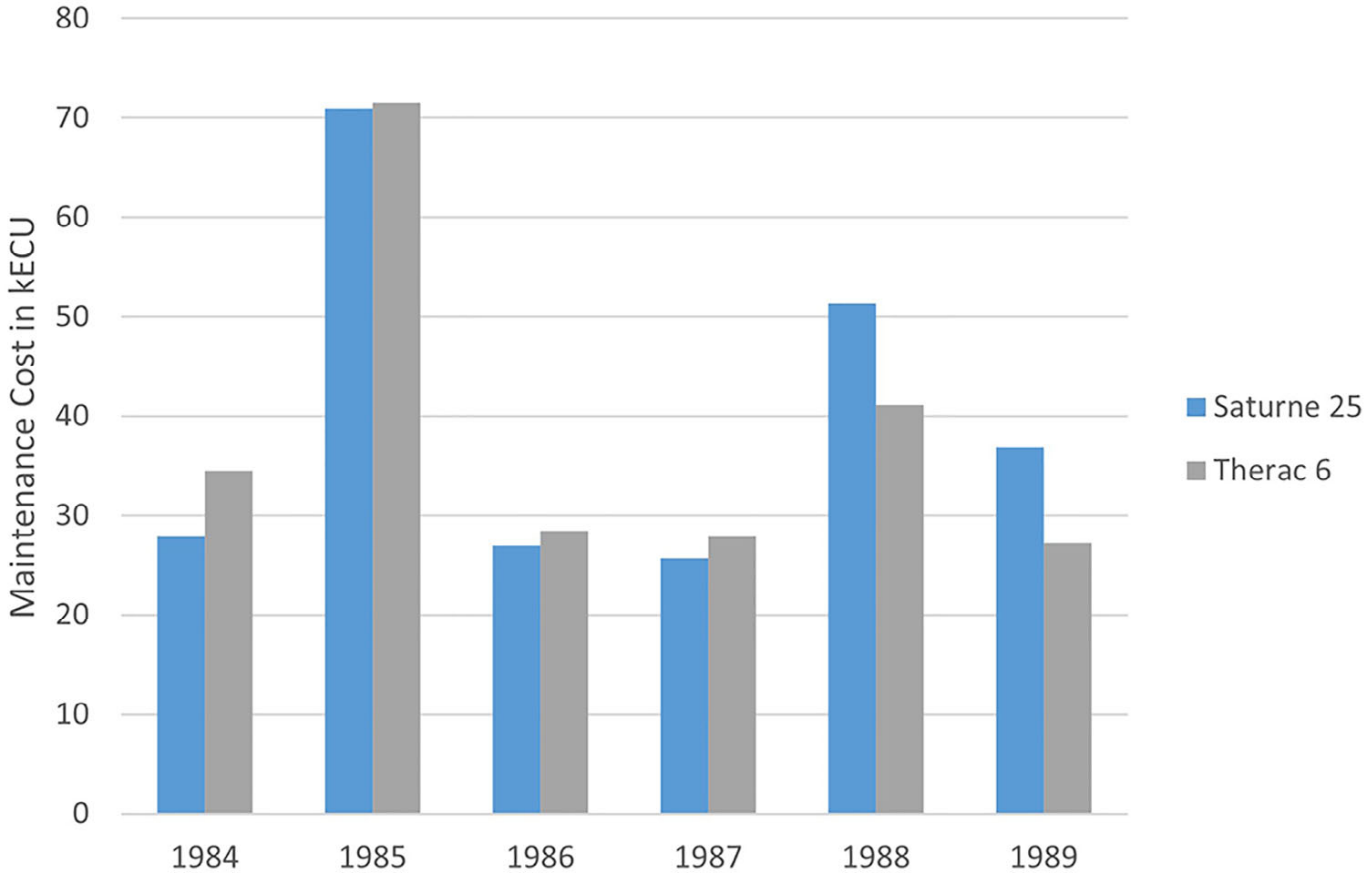
Adaptation of Figure 1A from reference^4^ (van der Giessen). The *Y*‐axis are costs in ECU, or European Council Units, which was the precursor to the Euro. The exchange rate was about 1.25 USD per UCU.

For medical linear accelerators, van der Giessen studied the maintenance costs of linear accelerators as compared to cobalt units.^4^, ^5^ This study was performed over a period of six years, and was in an older era (1980s), before VMAT and IGRT became standard practice in radiotherapy. The average maintenance costs for cobalt units were about 20,000 ECU (European Council Units, valued at $1.25 USD) less than for the medical linear accelerators studied. In addition, the maintenance costs were found to be quite variable on a year over year basis. The results were not expressed as a percentage of the acquisition cost, so comparison to modern practice is difficult, nor did they report the statistics of their results, however it appears that the average maintenance costs were about 20,000 to 40,000 ECU per year. Their principal results are reproduced here in Figure 1 for the convenience of the reader. The same author also studied the general costs of radiotherapy in different parts of the world, which included a survey of maintenance costs.^6^ They found a large range of linear accelerator annual maintenance costs, from between $3,000 to $91,740 USD, with a median of $41,390 USD. Van Dyk and colleagues conducted a study examining the various costs in radiotherapy delivery and looked at factors such as country and department size.^7^ In their analysis, they used a 10% service cost ratio for equipment maintenance. Finally, Ploquin and Dunscombe performed a literature review summarizing the understanding of the cost structure for radiotherapy treatments.^8^ Their analysis did not discuss linear accelerator maintenance costs directly, however, they discussed the costs of “supporting clinical infrastructure” and reported literature values of about 20% of the total cost for radiotherapy per patient. They did not specify the components of supporting clinical infrastructure in their study so it is unclear how this relates to the specific machine maintenance costs for linear accelerators. They did report, however, a rising total cost for radiotherapy between the dates of the study (1981 – 2002), with costs approximately doubling during that time period.

Many radiotherapy facilities choose vendor service contracts in order to maintain devices; costs of the service contract to the end user and actual maintenance cost as born by the service contract provider are not generally known publicly, but are believed to be on the order of 6%−7% of the initial equipment cost*.

Expanding access to radiotherapy services is a global health problem. In a landmark study by Atun and colleagues,^9^ the global deficit of radiotherapy services was estimated at between $100B—$200B to meet radiotherapy needs between 2015 and 2035. Sung and colleagues estimate that the incidence of cancer is estimated to increase by 47% between 2020 and 2040.^10^ Finally, according to a study by Zubizarreta and colleagues,^11^ the number and distribution of medical linear accelerators in the world is on the order of about 10,000 to 15,000. Of these installations, roughly one third of the installations are in North America, one third in Europe, and one third in South East Asia and Australasia. They report that certain areas of the world including Africa and Latin America represent a disproportionate deficit in radiotherapy infrastructure, meaning that in order to meet global demand, these regions will bear a larger initial capital cost as well as ongoing maintenance costs.

Elekta discussed the linear accelerator requirements needed to address this upcoming demand in its 2018/19 annual report.^12^ The company estimated that the number of megavoltage machines needed to meet this need should increase by 8774 units between 2015 and 2035. Assuming a new machine cost of $3.5 M, and a service ratio of 6.5%, the annual maintenance costs alone for the required equipment would be about $2B USD. Since much of the need for additional radiotherapy capacity lies in lower and middle income economies,^11^ this implies that these regions will bear a large proportion of these costs.

Despite the large equipment costs involved, there are no modern studies of linear accelerator maintenance costs to our knowledge. Understanding achievable and accurate equipment maintenance costs would benefit all radiotherapy providers in both developed and developing economies since this information can be used to plan new facilities and provide realistic ongoing operating cost estimates. Our institution has a common radiotherapy program where maintenance, service contracts, IT, and other radiotherapy services are shared between 6 cancer centers with a total of 32 linear accelerators and one cobalt unit. The primary response to linear accelerator service issues is through in‐house service technicians that are situated on site. The purpose of this investigation is to draw from our experience in maintaining a large number of linear accelerators over multiple sites to investigate and understand the costs of servicing them and to report on the experience at our institution.

## 2 METHODS

Over a period of 675 days, from November 3^rd^, 2016 to September 2^nd^, 2018, all purchases of parts for the six sites of our institution and for medical linear accelerator repairs were manually entered into a database. The primary purpose of the database was to keep track of parts costs to justify the budget used for linear accelerator maintenance; a separate database was used to maintain service records. However, the database used in our study did include other information associated with linear accelerator service, such as the type of clinical equipment, the equipment serial number, a description of the problem, the solution to the problem, the name of the item purchased, the vendor, and the cost of the item purchased. Data was entered for all servicing done by the in‐house service groups, which included linear accelerators, but also CT scanners for radiotherapy simulation, orthovoltage equipment, cobalt teletherapy, brachytherapy equipment, as well as phantom and research equipment. The process ceased in the institution when an open purchase order (PO) process was initiated with the linear accelerator manufacturer, allowing parts from this manufacturer to be ordered directly by the service technician.

There were a total of 31 linear accelerators (all of which were manufactured by Varian) and one cobalt machine. During the time period of data collection, there was 1 linac replacement, so data was collected for a total of 33 distinct units. Of the linear accelerators, 15 were Varian Clinac™ type accelerators, and 16 were Varian TrueBeam™ type accelerators.

Since the data was entered manually by individuals from all six centers, the data was processed in order to reduce errors, and to further categorize the data. As well, since data was collected for all parts purchased within the radiotherapy departments, which included parts for non‐ linear accelerator equipment, the data was categorized to distinguish the equipment type. To separate the costs into appropriate categories, the Equipment type category was categorized as follows:

TrueBeam; Clinac; Clinical, non‐linac; Spare parts; Training; Door; Phantom; Facility; Software support; Treatment Planning System; IT Network.

The type “Clinical, non‐linac” mostly represented CT scanners, but also included orthovoltage equipment, Ultrasound equipment, brachytherapy equipment, C‐Arms, machine shop equipment, PET/CT, contrast injectors, and parts associated with the cobalt unit.

To limit the costs to equipment repair costs, the software support and treatment planning system costs were not included in the analysis. Costs were then grouped into costs per center per year. This was further divided into repair costs for Clinacs; TrueBeams; Clinical, non‐linac; and Spare parts. The total repair costs for each machine for the entire period were also computed. These were sorted from highest costs to lowest, and plotted as a cumulative chart. As well, the age of each linear accelerator was documented, and parts costs were plotted against machine age. Finally, the area of the machine where parts were used were identified, and parts costs were grouped by the machine area where the repair was performed.

Labor costs were also included in the study. Our institution employs linear accelerator service technicians who are responsible for maintaining all technical equipment within the cancer centers. To calculate labor costs, we used the maximum salary cost of the salary range of the job classification, and included benefits costs. Labor costs were calculated two ways. The first conservative way was to assume that all time was spent on linear accelerator servicing and use the total salary cost and divide by the number of linear accelerators to obtain a labor cost per machine. The second way was to calculate the proportion of parts related to linear accelerators, and assume that the labor cost was proportional to the proportion of parts used for linear accelerator servicing.

For the six centers in the study, there were a total of 20 service technicians employed during the study time. Multiplying by their average salary (including benefits), adding training costs†, and dividing by the number of machines (31) in the study yielded a labor cost per machine of $64,000. In the second method, where the labor costs were weighted by the ratio of parts used for linear accelerator repairs, the labor cost per machine was $40,700.

## 3 RESULTS

**FIGURE 2:**
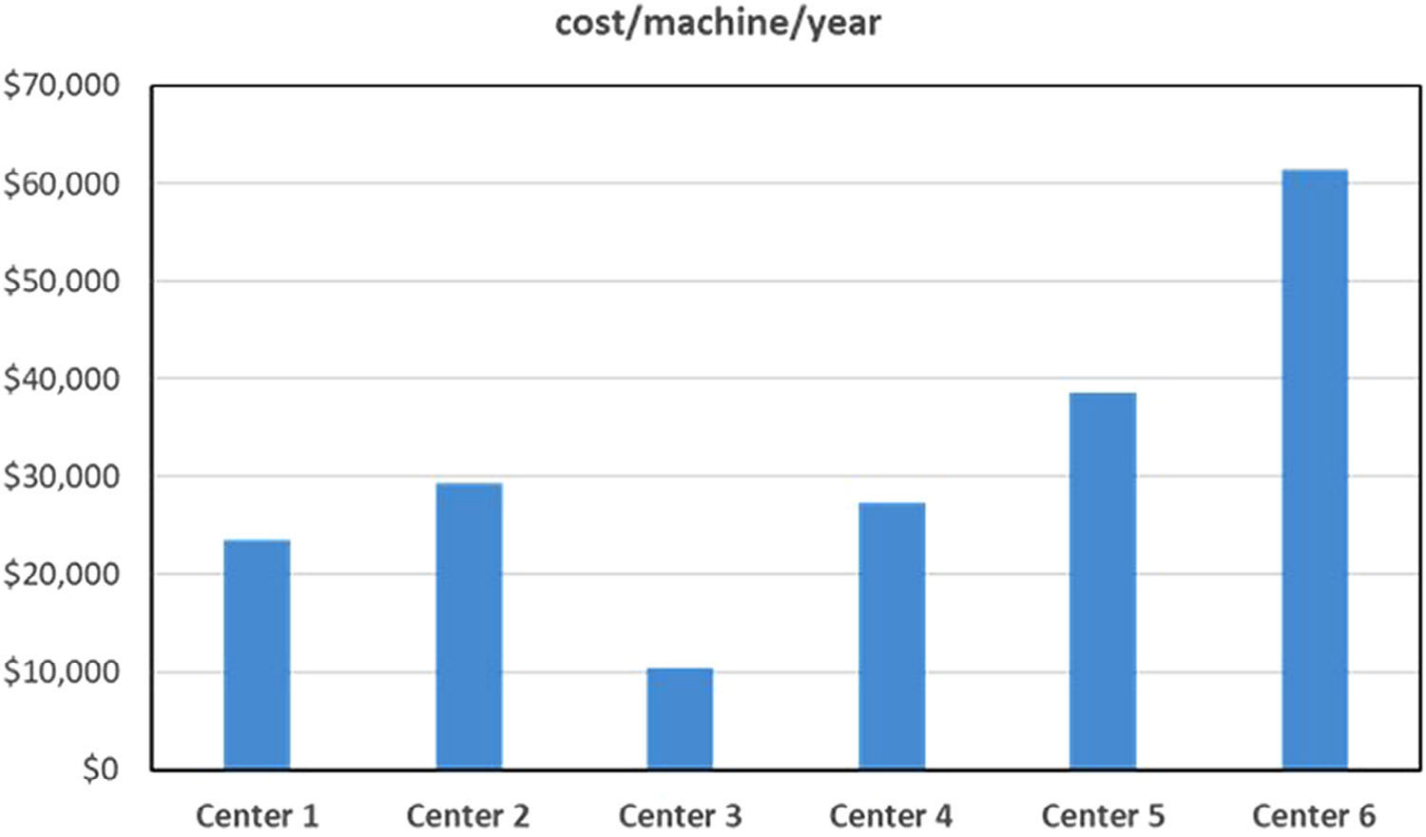
Total parts costs for each center, averaged over a 1 year period. Costs are in Canadian dollars.

The average cost of parts used per machine per year for machines grouped by the center where the machine operated are shown in Figure 2. The number of machines per center varied from 2 to 9; the numbers plotted in Figure 2 show the average parts costs for machines within the center. The range was for a high of $61,400 CAD to a low of $8,900 CAD. The median was $31,500.

**FIGURE 3:**
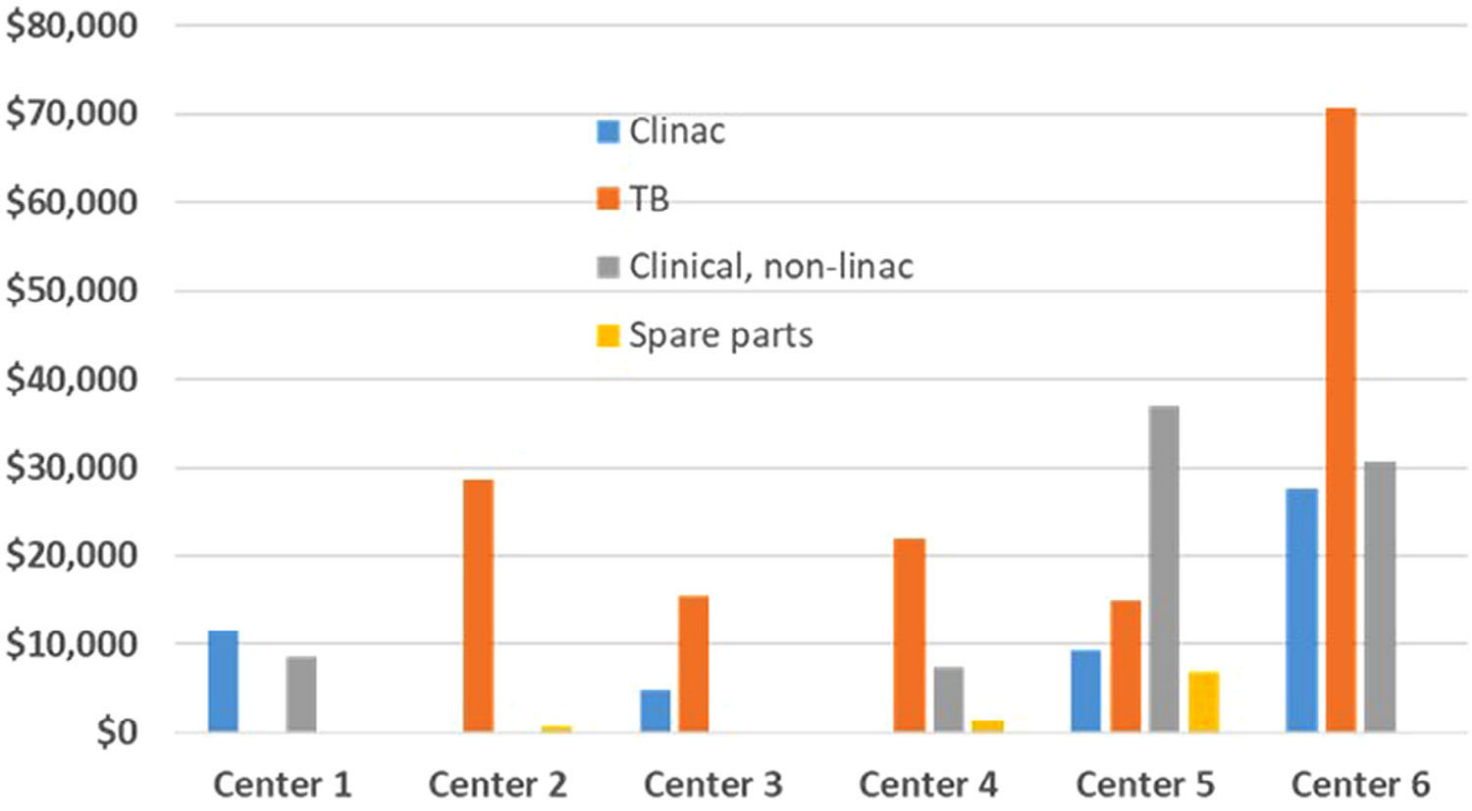
Parts costs for each center, separated into categories for machine type or spare parts, averaged over a 1 year period. Costs are in Canadian dollars. Centre 6 had 2 TrueBeam accelerators and 3 Clinac accelerators. These two TrueBeam accelerators had large parts costs, higher than the average of the 5 units at that center shown in Figure 2.

These costs were broken down into the categories Clinac, TrueBeam, Clinical—non linac, and spare parts. The breakdown of costs are shown in Figure 3. Two centers only had one equipment type (Centre 1 did not have any TrueBeam linear accelerators, and center 2 did not have any Clinac linear accelerators), and so for these centers, those data are not included.

For Clinac linear accelerators, the cost of parts ranged from a low of $4,750 CAD to a high of $27,600 CAD. The median parts costs was $13,300. For TrueBeam linear accelerators, the range was from a low of $11,600 CAD to a high of $70,600 CAD, with a median of $29,600. For non‐linear accelerator clinical units, the range was from a low of $0 to a high of $66,400 CAD, with a median parts cost of $17,000. The spare parts costs were lower, and amounted to an average of $1,500 CAD per machine for all six centers.

**FIGURE 4:**
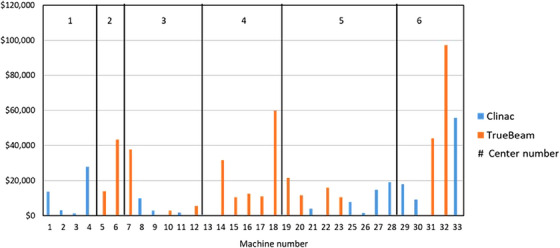
The total parts costs for each linear accelerator included in the study, separating Clinac units from TrueBeam units. The linear accelerator in the study were assigned a study number, which is plotted on the *x*‐axis. Costs are in Canadian dollars.

The cost of parts used for each linear accelerator is plotted in Figure 4, and separated into Clinac and TrueBeam linear accelerators, as well as the home center for the equipment. For Clinac linear accelerators, the parts costs ranged from a low of $1,450 CAD to a high of $55,800 CAD, with an average of $11,200. For TrueBeam linear accelerators, the low parts cost was $0 CAD, the maximum was $97,300 CAD, with an average of $21,500. We did not observe a correlation between maintenance costs per machine and the number of machines per center.

**FIGURE 5:**
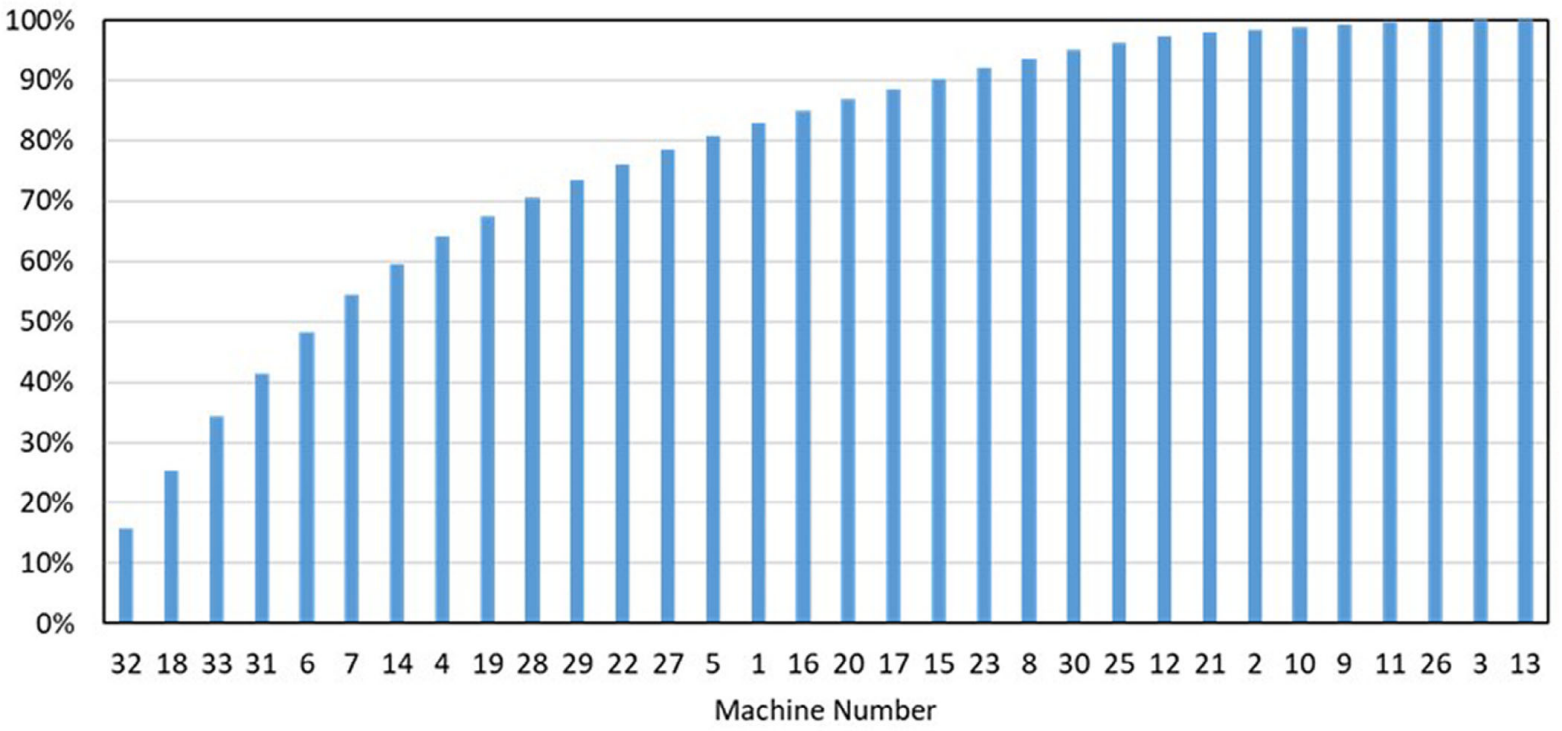
Parts costs, expressed as a percentage of the total parts costs, plotted from largest to smallest on a cumulative plot. From this plot we can see that 80% of the parts costs were attributed to 14 of the 33 linear accelerators.

In Figure 5, we plotted the cost of parts for each machine (from Figure 4) in order of decreasing cost on a cumulative plot so that the linear accelerators that contributed to the largest percentage of total costs could be identified. Each machine cost was normalized to the total cost. From this plot, we see that 14 of the 31 units contributed to 80% of the total costs.

**FIGURE 6:**
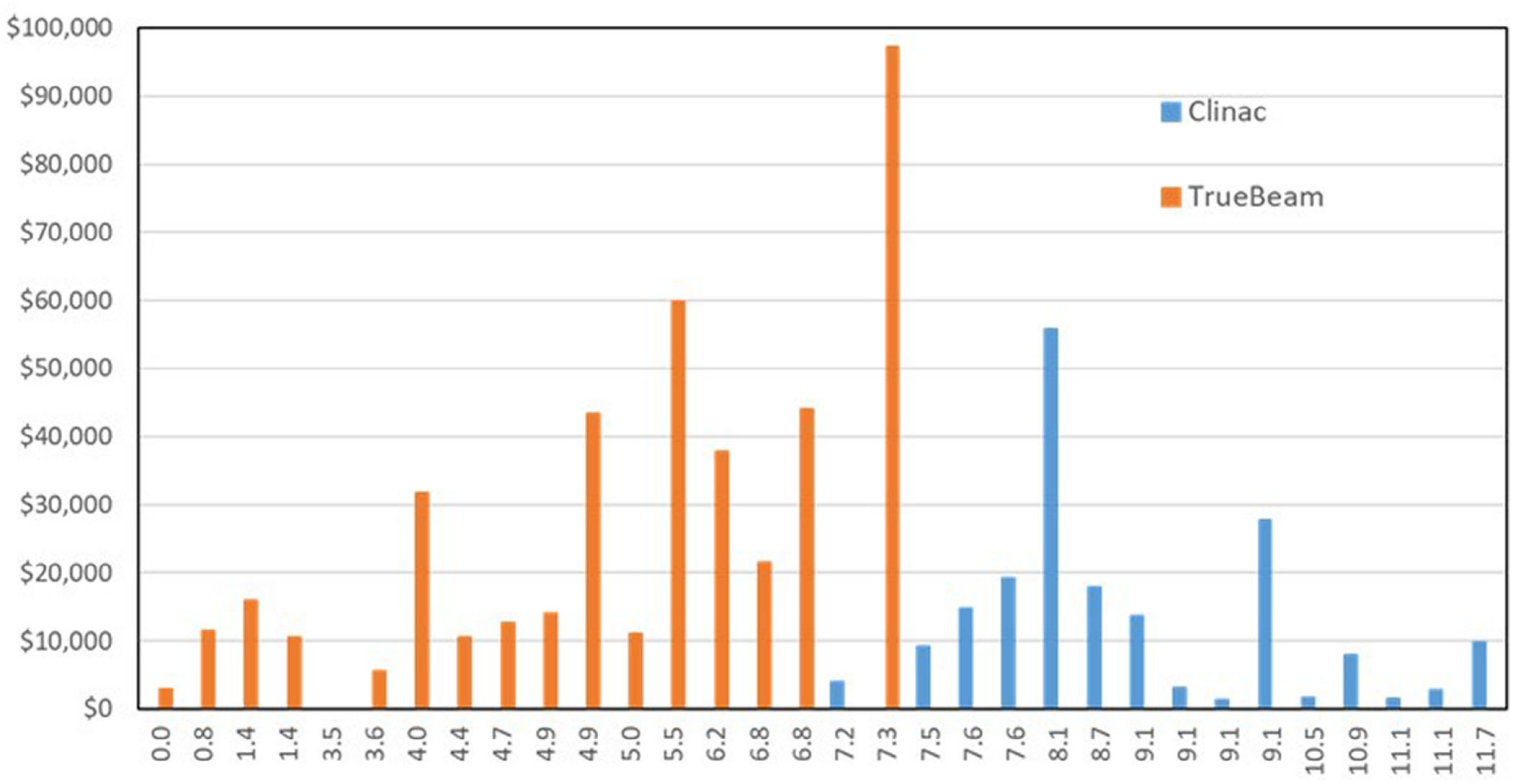
Parts costs, plotted against the median age of the linear accelerator at the time of the study. On the x‐axis, the median age is newest to oldest going from left to right. The older linear accelerators were Clinac style machines, while the newer machine (purchased from about 2009 onward) were TrueBeam style accelerators.

The machine install dates were used to calculate the mean age of each of the linear accelerators included in the study. The mean age was defined as the age of the machine at the average date of the study, which was the halfway date between the beginning and end date of the study. The parts cost for each machine plotted against the machine age is shown in Figure 6.

The parts were classified by the relevant area of the linear accelerator. The area classifications that were used are summarized in Table 1. In Figure 7 we plotted the costs associated with each of the areas in order of decreasing cost, expressed as a percentage of the total parts costs. These were divided into three plots, the firsts where all linear accelerators were included, the second for Clincac style only machines, and the third for TrueBeam style only machines.

**TABLE 1:** Area of the linear accelerator used to classify parts.

kV	Energy switch	Laser
Ion chamber	Gun	Power supply
Couch	Air cooling	Motor
MLC	Water cooling	Nodes
Labor	RPM	Field light
PCB	Covers	Carrousel
Thyratron	Mechanical	PMI parts
Pendant	High voltage electronics	Vacuum
MV	Target	Klystron
Low voltage	Jaw	Lightbulb
Console	Fasteners	

FIGURE 7Plots of component cost for all machine types (top), Clinac only machine types (middle) and TrueBeam only machine types (bottom). A demarcation is added to mark the component areas that contribute 80% of the machine costs. The areas that are present in the top 80% of the costs for all three plots are kV, MV, Thyratron, ion chamber, MLC and PCB (printed circuit board).

**Figure:**
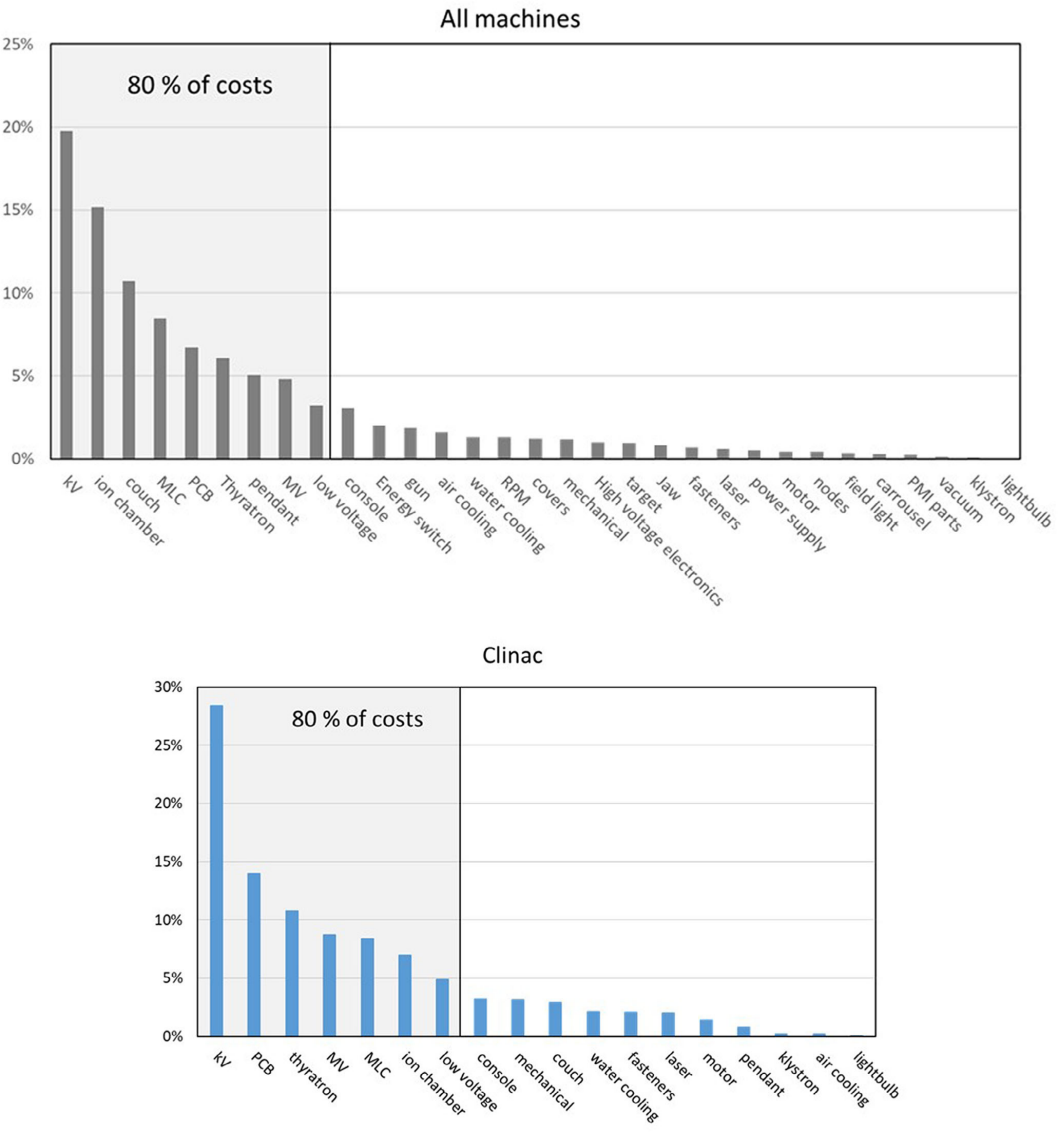

**Figure:**
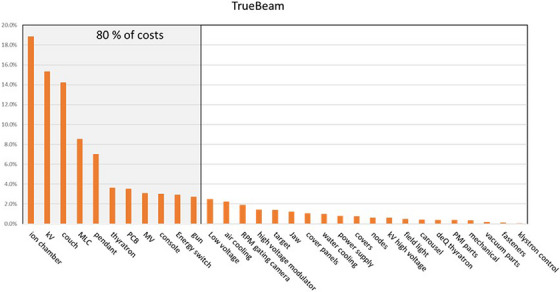

To estimate the maintenance cost ratio, we took the total parts costs for Clinac and TrueBeam linear accelerators and added spare parts costs and calculated the yearly average. We divided this by the total number of linear accelerators in the study to obtain an average parts cost per linear accelerator, which came to $41,600 CAD. To this we added the labor cost, which were calculated above. Including labor, the total maintenance cost per machine was $94,000 [$82,300, $105,700,].

Assuming an average purchase price of $3,000,000 CAD per linear accelerator, the average maintenance cost to capital cost ratio (excluding software support expenses) was 3.13%, [2.74%, 3.52%,]. This ratio, when calculated for individual linear accelerators ranged from a maximum of 4.99% [4.60%, 5.25%,] to a minimum of 1.75% [1.35%, 2.14%,], with the lower value approaching the cost of labor for service technicians.

## 4 DISCUSSION

The objective of this work is to estimate the service ratio cost for medical linear accelerators using our experience of the large number of medical linear accelerators in use at our institution. As with previous studies, we found this value to be variable from machine to machine, varying roughly between 2% to 5%, with an average of just over 3%. Of this cost, about 2/3 can be attributed to the labor cost we incurred at our institution. We did not include cost of the facility or insurance costs, as these were not available to the authors.

The labor costs at our institution may not be representative of other areas in the world. Canada is a High Income Country (HIC), with higher labor costs than other areas of the world. The results presented here are only applicable in high income countries, and may not be applicable in areas of the world where new linear accelerator infrastructure is needed, such as in lower income countries (LIC). It is beyond the scope of the current work to thoroughly investigate the service cost ratio in different geographic regions of the world, however to understand the difference in this ratio globally, we have used some data compiled by Van Dyk and colleagues.^7^ In their analysis, they estimated the labor costs and training costs for radiotherapy staff in both HICs and LICs. Using the data compiled in their Table 1, the training cost in an LIC is about 1/5 (22%) of the cost in an HIC, and the labor cost is about 1/20 (4.1%). Using these numbers to adjust our labor costs, and assuming a lower capital cost of $2,000,000 for the linear accelerator, the service ratio becomes 2.2% percent [2.18%, 2.23%]. However, for the LIC, the dominant cost is the cost of parts, which contribute 90% of the total costs. These results are only a rough guide since there are many variables that affect this result, such as the price of linear accelerators in LICs and potentially different parts prices. As well, there are other barriers to linear accelerator servicing in LICs such as import issues which are beyond the scope of this investigation. However, this illustrates that the service ratio estimate that we found at our institution may not be applicable in all areas of the world.

For HICs, our estimates are on the lower end of the studies for which we found some previous data, where the service cost ratio varied between 4.36% and 7.34%. Our result of 3.13% [−1.38%, + 1.86%,] is distinctly lower than previous estimates. We cannot say if this result is statistically significant since we do not have access to the statistics of the previous study. However, as in previous studies, we did see variability of service ratio costs, but the order of the variability was only about 1 – 2%. Contributing to the lower variability of our results is the labor cost, which was the largest contributor to the service ratio cost, but also the most stable, which minimized the cost variability machine to machine.

In our study, we had a large number of machines (over 30), and we collected data for almost a two year period. To our knowledge, this is the largest sample size for a maintenance cost analysis of medical linear accelerators. We observed differences between parts costs for older Clinac linear accelerators, and newer TrueBeam linear accelerators, with the older Clinacs less costly than the newer TrueBeams. From Figure 7, there were a larger number of areas of failure for TrueBeams than for Clinac style linear accelerators. As well, the cost of parts ordered for TrueBeam couch repairs was notably larger than for Clinac couch repairs, however the cost difference does not explain the entire cost discrepancy between TrueBeams and Clinacs.

The parts that contribute to the largest proportion of costs are identified in Figure 7. The list of components covers usual linear accelerator parts and include expected items such as imaging panels. The kV panels contributed more parts costs than the MV panels. For non‐imaging equipment, the ion chamber, thyratron, MLC components stand out as parts that has a high cost, and so were used more often. Other parts that were replaced often included components from most areas of the machine, meaning that no one single area contributed to a large proportion of parts costs. Wroe and colleagues^13^ as well as Sheehy and colleagues^14^ also studies failure modes in medical linear accelerators and used a similar classification scheme, but with less detail than the one that we developed in Figure 7 and Table 1. The focus of their work was to understand the medical linear accelerator failure modes in Lower and Middle Income Countries (LMIC), as opposed to the UK, to better understand the components that contributed to failure rates. Neither group studied the costs of the failures.

Absent from our list of parts is a klystron, which is a high cost part, however, we did not observe a klystron failure in the units included in our study, and over the time period of the study. There were a total of two components ordered for klystron related repairs, but these were components that interfaced with the klystron, not the klystron itself. It is not clear if this result is generally applicable, however, it is known that klystrons have a long life expectancy, and so it is reasonable that this part did not contribute to our largest costs. Magnetrons are also absent, however, none of the linear accelerators included in the study were magnetron powered, so this component would not come up in our study.

Comparing our results to the work of van der Giessen, the absolute dollar costs of parts is distinctly lower that the costs reported in his results, even though the current study was conducted about 35 years afterwards. In his Figure 1A (adapted and represented here as Figure 1), parts costs for the six machines studied ranged from about 30,000 to 70,000 ECU (the precursor to the Euro), which when converted to Canadian dollars (the currency used in this study) is about $50,000 CAD to $125,000. This is significantly higher than the costs we measured, $13,300 for Clinac linear accelerators, and $29,600 for TrueBeam linear accelerators. From Table 1 and Figure 7, we further see many components contributing the largest proportion of parts costs in the present study were not used in linear accelerators relevant to the van der Giessen study, as these machines would have predated the era of MV panels, kV imaging and MLCs. Removing these parts costs from our study to compare more directly with van der Giessen's results, it appears as though the cost of parts is substantially lower during the time period of this study than during the time period of van der Giessen. This result is different from the overall cost increases reported by Ploquin and Dunscombe.^8^ The reasons for this are not clear from the data collected in this study, however they may related to the generally improved manufacturing methodology available for modern linear accelerators than for those studied in van der Giessen's time period. Regardless of the reason, our results suggest that parts costs for linear accelerator maintenance may be lower than the industry may expect based on previous studies.

When plotted against machine age, which is shown in Figure 6, the results indicate that repair costs may peak for linear accelerators that are about 7 years of age and then decline afterwards. However, in this dataset the linear accelerators that are newer than 7 years are all TrueBeams, while the linear accelerators that are older are all Clinacs. The lower parts costs for Clinac linear accelerators may simply be a reflection of the age of these machines as opposed to an inherent difference in the servicing costs of the two units. It is also unclear if this data trend would apply to other linac models. Although it is difficult to draw firm conclusions from this relatively small data set due to the variation in the data and the machine models, if costs do in fact peak around the 7 year mark, this may have important implications on machine replacement cycle strategies and maintenance cost projections. Our study was not designed to measure the effect of machine age on linac maintenance costs; such a study should isolate equipment type, and have sufficient machine numbers within the study to be robust to any outlier data points. We suggest, however, that this is an interesting trend that may be important to the radiotherapy community, and that more study is needed to better understand the effect of machine age on service costs.

For our institution, the primary contributor to linear accelerator service costs was labor costs of service technicians. The absence of qualified service technicians would therefore present upward cost pressure on linear accelerator maintenance. While we didn't study the effect of training, the availability of service technicians for linear accelerators (a complex medical device), imply a certain level of base training, which can be attributed to lower maintenance costs than service contracts, which are about 6% ‐ 7% of capital costs for medical linear accelerators. With an average cost of 3%, we see a cost reduction on the order of 50%.

## 5 CONCLUSION

The service costs for 32 medical linear accelerators were studied over a two year period. It was found that the service cost as compared to capital equipment purchase cost was 3.13% [+ 1.86%, −1.38%]. This result is lower than other results previously reported for biomedical equipment, and linear accelerators specifically. We found that the service cost was variable machine to machine, indicating that service costs should be expected to fluctuate year over year. We also found that service cost were maximum for linear accelerators of about 7 years of age, with service cost being lower for newer and older machines. The largest contributor to the service costs was labor, which consisted of about 2/3 of the cost of the equipment maintenance.

## AUTHOR CONTRIBUTIONS

Marco Carlone contributed to the collection of data, performed the data analysis, wrote the first version of the draft manuscript, and coordinated the review from the co‐authors. Wayne Beckham contributed to the study design, to the collection of data and reviewed the manuscript. Cheryl Duzenli contributed to the collection of data and reviewed the manuscript. Kirpal Kohli contributed to the collection of data and reviewed the manuscript. Scott Tyldesley contributed to the study design, to the collection of data and reviewed the manuscript.

## CONFLICTS OF INTEREST STATEMENT

Marco Carlone is the founder and majority owner of Linax Technologies Ltd, 1869−3151 Lakeshore Rd, Kelowna, BC V1W 3S9.

## Footnotes

* Based on a service contract cost of $220,000 for a $3.5M new medical linear accelerator in 2023 prices.

† Training costs for the period of the study were $52,000 for two factory training courses. Junior service technicians go through a two‐year apprentice program, which was included in this study as labor costs.
